# Madelung's Disease: Lipectomy or Liposuction?

**DOI:** 10.1155/2018/3975974

**Published:** 2018-02-21

**Authors:** Chun-Ye Chen, Qing-Qing Fang, Xiao-Feng Wang, Min-Xia Zhang, Wan-Yi Zhao, Bang-Hui Shi, Li-Hong Wu, Li-Yun Zhang, Wei-Qiang Tan

**Affiliations:** ^1^Department of Plastic Surgery, The Fourth Affiliated Hospital, College of Medicine, Zhejiang University, Yiwu, Zhejiang, China; ^2^Department of Plastic Surgery, The First Affiliated Hospital, College of Medicine, Zhejiang University, Hangzhou, Zhejiang, China

## Abstract

**Background:**

Madelung's disease is a rare lipid metabolic disorder characterized by diffuse, uncapsulated lipomas in the neck, shoulder, and other areas. It mainly affects middle-aged men and is related to alcohol abuse, and the cause is not clear. Surgical treatments include lipectomy and liposuction.

**Methods:**

This systematic review analyzed the treatment of Madelung's disease described in 52 articles including complete patient details, published between 2000 and 2015, and retrieved from the Web of Science, PubMed, Medline, and Embase.

**Results:**

Lipectomy was performed in most cases and achieved more complete removal and better control of iatrogenic lesions of nearby structures than liposuction. Liposuction achieved good cosmetic results and is simpler and less invasive than lipectomy, but clinical experience is limited.

**Conclusions:**

Both lipectomy and liposuction have advantages and drawbacks. Surgeons should base the choice of optimal treatment on patient characteristics. Novel surgical techniques and etiologically targeted treatments hold promise as future therapies.

## 1. Introduction

Madelung's disease, multiple symmetrical lipomatosis, or benign symmetric lipomatosis is a rare disorder of adipose metabolism that was first reported in 1846 by Benjamin Brodie. It presents with multiple, symmetrical, nonencapsulated fatty masses in the maxillofacial region, neck, shoulder, trunk, limbs, and other areas. In most patients, neck involvement results in a specific sign called Madelung's or horse collar [1, 2]. Madelung's disease is more common in middle-aged men of Mediterranean origin and is related to alcohol abuse. However, cases have been reported in women, patients in south east countries, and in nondrinkers [1–3].

The disease is not painful; most but not all cases have a slow clinical course [1], and malignant transformation is infrequent [1, 2]. Transformation to intramyxoid sarcoma after a 6-year follow-up was reported by Tizian; a case of liposarcoma transformation was reported by Sia et al. [3–5]. Cosmetic deformity and compression symptoms such as dyspnea and dysphagia are indications for treatment. Comorbidities such as liver cirrhosis, nephropathy, neuropathy, and metabolic disorders have been reported [1–3], but no causal relationships between those disorders and Madelung's disease have been established [6]. Two types of Madelung's disease can be distinguished by their clinical characteristics [1, 2, 6]. Type I occurs most often in men, generally as a symmetrical distribution of superficial fatty masses with a “pseudoathletic” appearance, which are likely to cause compression symptoms. Type II can occur in both men and women, with a presentation similar to generalized obesity. The classification is not very exacting.

The etiology of Madelung's disease has not been established, but recent evidence has implicated mitochondrial dysfunction resulting from mutations of mitochondrial DNA [1, 2, 7] and changes in enzyme and membrane receptor activity that reduce adrenergic-mediated lipolysis. Alcohol consumption might serve as a predisposing or aggravating factor [6]. The diagnosis can made by clinical features and imaging studies [2]. Computed tomography, ultrasonography, and magnetic resonance imaging determine the adhesion to, size, location, and compression of adjacent tissue. Imaging thus helps with the diagnosis, differential diagnosis, and preoperative assessment [2, 8]. The use of magnetic resonance imaging for assessment and preoperative planning has been recently reviewed in a case report by Sharma et al. [9]. Histologically, the adipocytes present in the fatty masses in Madelung's disease are smaller, and the content of fibrous and vascular tissues is greater than normal [6]. There is also a loss of large myelinated cells, but without the demyelination or axonal degeneration induced by chronic alcohol ingestion [2]. At least one report found that the triglyceride content of fat cells was affected [6]. This systematic review summarizes the current treatment of Madelung's disease and the choice of treatment in different clinical contexts.

## 2. Methods

### 2.1. Literature Search

This systematic review followed the PRISMA guidelines. The Web of Science, PubMed, Medline, and Embase were searched for case reports of Madelung's disease published from 2000 to 2015. The search terms were “Madelung disease,” “Madelung syndrome,” “multiple symmetrical lipomatosis,” and “benign symmetric lipomatosis.” Duplicate publications, meeting abstracts, treatment guidelines, recommendations, literature reviews without case reports, experimental animal model studies, etiologic investigations, and cases reports without clear clinical data including patient age, sex, symptoms, and treatment were excluded.

### 2.2. Analysis

A total of 52 studies published from 2000 to 2015 included complete patient characteristics and treatment descriptions and were selected for review [1–3, 8, 10–57]. The studies are listed in Table 1. For ease of understanding, the clinical characteristics and treatment methods of these patients with Madelung's disease are summarized in Table 2.

## 3. Results

### 3.1. Characteristics of Reported Cases

The average age of the 106 reported cases from the 52 studies (Table 1) was 52.9 years, and the male-to-female ratio was 93 : 13. The majority of masses were located on the neck and face, but the trunk and limbs are also common sites. Lipomas were rarely reported around the eye or on the tongue, throat, breast, pubis, and testis. The most frequent comorbidities were nephropathy, hepatopathy, and metabolic abnormalities. Klinefelter syndrome, acquired immunodeficiency syndrome, and sexual dysfunction were rarely reported comorbidities.

Surgery was the most frequent treatment, 8.49% of the patients were treated medically. Intralipotherapy was used in three patients (2.86%) described in two reports. One intralipotherapy patient (Table 1a) had a recurrence 4 years after treatment. It was treated surgically without a second recurrence at 32 months of follow-up [10]. The other two patients (Table 1c) had both experienced a successful prior surgical excision [18]. And this study showed that intralipotherapy achieved a 2.5% reduction in average size and had a 33% recurrence rate [18]. Medical therapy was described in five reports. Lipomatosis remained unchanged in two of those patients (Table 1b) after 4 months to 3 years of follow-up [14, 32]. The other three studies did not report whether any relapses had occurred.

Ninety-five patients (89.62%) were treated surgically by lipectomy, and 79 of them were treated by lipectomy only. Four of those 79 patients experienced relapses, one experienced malignant transformation (Table 1f) [28], and 30 patients with reported follow-up did not experience a relapse. Whether or not relapse occurred was not reported for 24 patients. One surgery patient (Table 1d) had failed liposuction [20]. Only 18 patients (16.98%) were treated by liposuction, and 2 of them were treated by liposuction only: one experienced relapse after 1 year of follow-up [53], and the other case was not mentioned. Sixteen of the 18 patients experienced procedures that were combined with lipectomy (Table 1e) [23, 43, 47, 54, 56]. Two of the 16 patients experienced relapse after 1 and 1.5 years of follow-up [49].

### 3.2. Synthesis of Results

Table 2 summarizes the clinical characteristics of the published cases of Madelung's disease. Most patients were middle-aged men, and the most frequent locations were on the neck and face. The most frequent comorbidities were nephropathy, hepatopathy, and metabolic abnormalities. Most patients were treated surgically by lipectomy and a few by liposuction. Medical procedures were seldom used and included mainly intralipotherapy. Novel techniques included combined lipectomy and liposuction, ultrasound-assisted liposuction, tunneling techniques, cervical dissection techniques, powered-assisted liposuction, and a rhytidectomy approach. The main treatment-related problem was relapse, especially following liposuction.

## 4. Discussion

Treatment for Madelung's disease included both surgical and nonsurgical procedures. Surgical treatment was the most frequent treatment and included lipectomy and liposuction.

### 4.1. Nonsurgical Treatment

Some reports recommended intralipotherapy as a noninvasive treatment of Madelung's disease, in which the mass was injected with phosphatidylcholine/deoxycholate. Scevola et al. confirmed the efficacy of intralipotherapy after long-term assessment [18]. Intralipotherapy mainly limits the growth of adipose masses and is not highly effective in reducing their volume. If surgical dissection is planned, then surgeons should be aware of the potential for adhesions in patients who were previously treated with injection lipolysis [10]. Abstinence from alcohol may slow the enlargement of adipose masses but cannot reverse the disease. The risk of recurrence is decreased by abstaining from alcohol after surgery. Other lifestyle modifications that improve blood glucose and lipid control in patients with comorbidities may prevent growth of adipose masses but do not reduce their size [3]. Dietary management does not help [2]. Other therapeutic methods such as injecting lesions with enoxaparin and *β*2 agonists [2] may be effective, but their curative effects have not been clearly demonstrated.

### 4.2. Lipectomy

Lipectomy and liposuction are the most effective surgical options, but because the etiology of Madelung's disease is not clear, they are seen as palliative surgery [3]. Lipectomy was performed in most reported cases, allowing for satisfactory exposure and allowing for complete removal with good control of iatrogenic lesions of nearby structures, especially vessels and nerves. Although direct visualization reduces the risk of injury, lipectomy is not technically easy because the lipomas are nonencapsulated with wide infiltration of surrounding tissue [2, 58]. During surgery, the pathological hyperplastic lipoma is difficult to distinguish from subcutaneous fat and other healthy tissues [3]. Lipectomy has disadvantages of an increased rate of surgical complications including infection, hemorrhage, hematoma, lymphatic fistula, and pathologic scarring [1, 2, 8, 58]. Although it is an open approach, debulking surgery has been recommended as allowing for a better cosmetic result than lipectomy, with the exception of scar formation [6]. Most reports recommend a single transverse incision rather than multiple direct incisions to remove Madelung's collar [2].

### 4.3. Liposuction

Liposuction has gained in popularity because it is less traumatic and has better cosmetic outcomes than surgery. However, because it has not been widely used, there is little clinical experience with liposuction in Madelung's disease patients. The advantages of liposuction include simplicity, minimal invasiveness, and low morbidity; it has been an adjunct therapy in many cases. In addition, its dense and fibrous composition make it difficult to aspirate the fatty tissue in the conventional way [1, 2, 8], posing a problem for surgeons during liposuction. The nature of the adipose masses also decreases the reliability of preoperative assessment of possible liposuction failure. There are no histological assessments, like the extent of fibroplasia, that allow avoiding the difficulties of liposuction [8]. It is difficult to achieve complete removal either by liposuction or lipectomy because the lipomas are nonencapsulated, making recurrence nearly unavoidable. Finally lipectomy and liposuction are not simple because growth of the fatty masses may proceed in more than one direction and involve several structures [6].

### 4.4. Indications for Surgical Treatment

As there is no definitive, etiologically targeted treatment, all treatments are palliative. The goal of treatment is recovery of function and improved appearance. It is not necessary to place patients at risk by performing premature, radical surgery for this benign disease [3]. Lipectomy is considered the treatment of choice for Madelung's disease, performed primarily in severe cases involving multiple body structures or producing compression symptoms [1]. Lipectomy is necessary if there is obstruction of the trachea or pharynx leading to dyspnea, dysphagia, or related symptoms [1]. Lipectomy is also indicated for treating severe deformities and for removal of redundant skin [8]. Liposuction has been used to treat less severe cases with limited areas of involvement or as a second-stage or adjunct treatment. If the lipomas are very diffuse, making them difficult to remove by lipectomy, liposuction should be considered [6]. Liposuction is also more suitable for patients at increased surgical and/or anesthetic risk [3]. Some authors recommend that liposuction not be applied in the submental region or in regions with scars from previous surgery [8]. Grassegger et al. reported the safe use of tumescent liposuction in patients with Madelung's disease and liver cirrhosis. The patients had compensated disease, minor procedures, low total lidocaine doses, and careful postoperative surveillance [7].

### 4.5. Novel Techniques

Some revisions of standard lipectomy and liposuction treatment have been evaluated in Madelung's patients. In 2003, Constantinidis et al. reported satisfactory outcomes using combined lipectomy and liposuction in 11 cases with masses in the head and neck region [49]. Bassetto et al. described use of ultrasound-assisted liposuction to prevent injury to surrounding structures, such as blood vessels and nerves [58]. This technique provided gentle, precise liposuction that reduced damage to subcutaneous tissue and vascular structures but had unsatisfactory cosmetic outcomes [3, 58]. With the tunneling technique, most subcutaneous neurovascular plexus remain intact, and the problem of redundant loose skin is also solved [59]. Andou et al. reported a case in which improved lipectomy using a cervical dissection technique was helpful for treating Madelung's collar [11]. Tremp et al. found that tightening of the skin achieved by power-assisted liposuction improved cosmetic outcomes and was an effective method [60]. Pinto et al. proposed a rhytidectomy approach for recurrent Madelung's disease [61] using multiple-stage surgery. A small submental incision and slightly modified rhytidectomy incisions were made to access the anterior and lateral neck during the supine stage. A posterior neck incision was then made during the prone stage to optimize skin redraping and address the posterior neck hump [34]. Pinto et al. [61] used an inverted T shaped incision (sagittal and suprasternal incisions) successfully, which offers good exposure and allows for removal of skin excesses.

## 5. Conclusion

The most effective treatments of Madelung's disease were lipectomy and liposuction, both of which have advantages and drawbacks. Before surgery, patients should be informed of the risks and benefits of lipectomy and liposuction and be made aware of the risk of lipoma recurrence. The choice of surgical procedure depends on the disease extent, patient expectations, and the surgeon's experience. This systematic review included a relatively small number of cases. Some clinical data was missing, and some reports were of rare cases that were not truly representative. Despite these limitations, the examples of successful treatment of Madelung's disease provide alternatives for managing patients. Future trends in treatment of Madelung's disease would benefit from study of its etiology and pathogenesis. Experience with modified techniques of liposuction or lipectomy should be expected to make treatment more effective.

## Figures and Tables

**Table 1:** 41 literature sources of Madelung's disease from 2000 to 2015 with complete materials of treatment.

Published date	Authors	Cases	Male : female	Age or average age	Treatment			After treatment		
					Lipectomy	Liposuction	Other	Relapse (follow-up times)	No relapse (follow-up times)	Unknown
2015	Azuma et al.	1	1 : 0	74	1	0	0	0	0	1
2015^a^	Andou et al.	1	1 : 0	51	0	0	1 (intralipotherapy)	0	1 (4 years)	0
2015	Kang et al.	1	1 : 0	64	1	0	0	0	0	1
2015	Zielińska-Kaźmierska et al.	1	1 : 0	46	1	0	0	0	0	1
2015^b^	Ozderya et al.	1	1 : 0	29	0	0	1 (medical therapy)	0	1 (3 years)	0
2014	Jang et al.	1	1 : 0	66	1	0	0	0	0	1
2014	Celentano et al.	1	1 : 0	51	1	0	0	0	1 (36 months)	0
2014	Z. Pinnella and J. Pinnella	1	1 : 0	52	1	0	0	0	1 (30 months)	0
2014^c^	Scevola et al.	2	2 : 0	47	0	0	2 (intralipotherapy)	0	0	2
2014	Orasmo et al.	1	1 : 0	48	1	0	0	0	0	1
2014^d^	Hundeshagen et al.	2	2 : 0	58.5	2	0	0	0	2 (8–13 months)	0
2014	Tai et al.	1	1 : 0	54	1	0	0	1 (2 years)	0	0
2014	Shibasaki et al.	1	1 : 0	47	1	0	0	0	0	1
2014^e^	Agostini et al.	1	1 : 0	62	1	1	0	0	1 (1 year)	0
2013	Nikolić et al.	1	1 : 0	51	1	0	0	1 (3 years)	0	0
2013	Kang and Kim	1	0 : 1	76	1	0	0	0	0	1
2013	Ardeleanu et al.	1	1 : 0	55	1	0	0	0	0	1
2013	Klobucnikova et al.	2	2 : 0	58	2	0	0	0	0	2
2012^f^	Borriello et al.	1	0 : 1	59	1	0	0	1 (malignant)	0	0
2012	Lee et al.	1	1 : 0	50	1	0	0	0	1 (10 months)	0
2012	Sia et al.	1	1 : 0	45	1	0	0	0	1 (1 week)	0
2012	Subash et al.	1	1 : 0	49	1	0	0	0	0	1
2012	Friedl et al.	1	0 : 1	55	0	0	1 (medical therapy)	0	0	1
2012^b^	Tufan et al.	1	1 : 0	56	0	0	1 (medical therapy)	0	0	1
2012	Mevio et al.	3	3 : 0	59.3	3	0	0	0	0	3
2012	Di Candia and Cormack	1	1 : 0	34	1	0	0	0	0	1
2011	Albu	1	1 : 0	59	1	0	0	0	1 (2 years)	0
2011	Lee et al.	1	1 : 0	71	1	0	0	0	1 (2.4 years)	0
2011	Ampollini and Carbognani	1	1 : 0	45	0	0	1 (medical therapy)	0	0	1
2010	Milisavljevic et al.	1	1 : 0	58	1	0	0	0	1 (1 year)	0
2009	Juric and Carapina	1	1 : 0	69	0	0	1 (medical therapy)	0	0	1
2009	Alameda et al.	1	1 : 0	55	1	0	0	0	0	1
2009	Tekin and Ogetman	1	1 : 0	26	1	0	0	0	0	1
2009	Lee et al.	1	1 : 0	64	1	0	0	0	0	1
2008	Verna et al.	4	2 : 2	55.25	3	1	0	0	0	4
2008^e^	Tan and Ergen	1	0 : 1	20	1	1	0	0	1 (22 months)	0
2007	Bulum et al.	1	1 : 0	51	1	0	0	0	0	1
2007	Ali and Kishore	1	1 : 0	53	1	0	0	0	1 (6 months)	0
2006	Lopez-Ceres et al.	1	0 : 1	57	1	0	0	0	0	1
2006^e^	Conroy	1	1 : 0	51	1	1	0	0	0	1
2005	Colella et al.	1	1 : 0	44	1	0	0	0	0	1
2004	Uglešić et al.	1	1 : 0	53	1	0	0	0	1 (5 months)	0
2004	González-García et al.	2	2 : 0	51.5	2	0	0	0	2 (1 and 10 years)	0
2003^e^	Constantinidis et al.	11	10 : 1	47	11	11	0	2 (1 and 1.5 years)	9	0
2003	Verhelle et al.	5	4 : 1	52.5	5	0	0	1 (10–48 years)	4	0
2002	Guastella et al.	2	1 : 1	59.5	2	0	0	0	1 (18 months)	1
2001	Ujpaál et al.	31	29 : 2	41.5	31	0	0	0	11 (17.1 years)	20
2001	Faga et al.	1	1 : 0	51	0	1	0	1 (1 year)	0	0
2001^e^	Nielsen et al.	1	0 : 1	50	1	1	0	0	0	1
2001	Fischer et al.	1	1 : 0	43	0	0	1 (intratumoral injection)	0	0	1
2000^e^	Payne	1	1 : 0	69	1	1	0	0	1 (1 year)	0
2000	Vargas-Díez et al.	1	1 : 0	59	1	0	0	0	1 (6 months)	0

Sum		106	93 : 13		95	18	9	7	44	55

Average				52.9						

Percentage					0.8962	0.1698	0.0849			

A total of 41 studies published from 2005 to 2015 included complete patient characteristics and treatment descriptions and were selected for review [1–3, 8, 10–57]. ^a^This patient had a relapse 4 years after treatment, then surgical procedure was performed, and there was no recurrence of the lipomatosis after a 32-month follow-up. ^b^The lipomatosis of 2 patients remained unchanged in 4-month to 3-year follow-up. ^c^These 2 patients both had successful previous surgical excision. ^d^This patient had failed liposuction before. ^e^Patients in these cases were submitted to liposuction combined with lipectomy. ^f^Malignant happened in this case.

**Table 2:** Summary of the clinic data of published cases of Madelung disease.

Age	Middle age

Sex	Appearing mostly in male

Most frequent locations	Neck and face

Most frequent comorbidities	Nephropathy, hepatopathy, and metabolic abnormalities

Treatment	
Surgical method (most)	Lipectomy (most) and liposuction
Medical procedure	Intralipotherapy and medical therapy
New techniques	Combined lipectomy and liposuction, ultrasound-assisted liposuction (UAL), tunneling technique, cervical dissection technique, powered-assisted liposuction (PAL), rhytidectomy approach, special incisions (a small submental incision and slightly modified rhytidectomy incisions, posterior neck incision, T shaped incision)

The main problem after treatment	Relapse, especially after liposuction

A simple summary of the clinic date of the 106 published cases of Madelung's disease.
